# Differences in N loading affect DOM dynamics during typhoon events in a forested mountainous catchment

**DOI:** 10.1016/j.scitotenv.2018.03.177

**Published:** 2018-03-21

**Authors:** Tz-Ching Yeh, Chien-Sen Liao, Ting-Chien Chen, Yu-Ting Shih, Jr-Chuan Huang, Franz Zehetner, Thomas Hein

**Affiliations:** a Institute of Hydrobiology and Aquatic Ecosystem Management, University of Natural Resources and Life Sciences (BOKU), Vienna, Austria; b WasserCluster Lunz (WCL), Inter-university Research Institute, Lunz am See, Austria; c Department of Civil and Ecological Engineering, I-Shou University, Kaohsiung, Taiwan; d Department of Environmental Science and Engineering, National Pingtung University of Science and Technology (NPUST), Pingtung, Taiwan; e Department of Geography, National Taiwan University (NTU), Taipei, Taiwan; f Institute of Soil Research, University of Natural Resources and Life Sciences (BOKU), Vienna, Austria

**Keywords:** Dissolved organic matter, Nutrient dynamics, Hydrologic extremes, Terrestrial-aquatic continuum, Typhoon event

## Abstract

The dissolved organic matter (DOM) and nutrient dynamics in small mountainous rivers (SMRs) strongly depend on hydrologic conditions, and especially on extreme events. Here, we investigated the quantity and quality of DOM and inorganic nutrients during base-flow and typhoon events, in a chronically N-saturated mainstream and low N-loaded tributaries of a forested small mountainous reservoir catchment in Taiwan. Our results suggest that divergent transport mechanisms were triggered in the mainstream vs. tributaries during typhoons. The mainstream DON increased from 3.4 to 34.7% of the TDN pool with a static DOC:NO_3_-N ratio and enhanced DOM freshness, signalling a N-enriched DOM transport. Conversely, DON decreased from 46 to 6% of the TDN pool in the tributaries and was coupled with a rapid increase of the DOC:NO_3_-N ratio and humified DOM signals, suggesting the DON and DOC were passively and simultaneously transported. This study confirmed hydrology and spatial dimensions being the main drivers shaping the composition and concentration of DOM and inorganic nutrients in small mountainous catchments subject to hydrologic extremes. We highlighted that the dominant flow paths largely controlled the N-saturation status and DOM composition within each sub-catchment, the effect of land-use could therefore be obscured. Furthermore, N-saturation status and DOM composition are not only a result of hydrologic dynamics, but potential agents modifying the transport mechanism of solutes export from fluvial systems. We emphasize the importance of viewing elemental dynamics from the perspective of a terrestrial-aquatic continuum; and of taking hydrologic phases and individual catchment characteristics into account in water quality management.

## 1 Introduction

Since the last decade, inland waters have been recognised as active sites processing organic carbon (Cole et al., 2007). About 2.7 Pg C yr^−1^ is transported, mineralised, and buried in inland waters, similar to the amount of the terrestrial C sink for anthropogenic emissions (2.8 Pg C yr^−1^, Battin et al., 2009; Tranvik et al., 2009). The source material and processing of dissolved organic matter (DOM) determine the fate of carbon (Hansen et al., 2016), which in fluvial systems is controlled by the interdependence between the spatial and hydrologic dimensions along the terrestrial-aquatic continuum (Ejarque et al., 2017). Building upon the idea of inland waters as active components of the global C cycle (Cole et al., 2007), the *Pulse-shunt Concept* further articulates that the catchments alternate between base-flow and episodic hydrologic events, such as snow melt or storms (Raymond et al., 2016). The drainage systems shift between instream process-dominant “*active pipes*” and export-dominant “*passive pipe*s” in terms of DOM reactivity, depending on factors which affect residence time (e.g. hydrology) and decomposition kinetics (e.g. temperature) (Raymond et al., 2016). DOM is the largest organic matter pool in riverine systems (Tank et al., 2010). This ubiquitous and complex source of organic compounds composed of both dissolved organic carbon (DOC) and nitrogen (DON) is closely linked to the ecosystem metabolism (Bertilsson and Jones, 2003; del Giorgio and Williams, 2005). In small mountainous streams (SMRs), the highly variable flow paths not only extensively determine the DOM characteristics (Yang et al., 2015) but also the magnitude of N export (Zhang et al., 2007) during extreme hydrologic events. However, to our knowledge, the interactions between riverine organic and inorganic C and N components during hydrologic extremes are rarely investigated.

Anthropogenic influences often act as the third dimension of disturbances on fluvial dynamics in addition to hydrology and longitudinal dimensions (Ward and Stanford, 1995). The influence of anthropogenic N enrichment (e.g. atmospheric-deposition, fertilization, etc.) on organic N loss has remained controversial (Pellerin et al., 2006). Several field studies suggested DON dominates over dissolved inorganic N (DIN) in N-limited forest ecosystems receiving low anthropogenic N inputs (Qualls, 2000; Perakis and Hedin, 2002; Park and Matzner, 2006; Schmidt et al., 2010). Dissimilarly, plot-scale N fertilization studies concluded that the majority of N is lost as DON from N-saturated systems (Brookshire et al., 2007; Fang et al., 2009), and that a higher labile DON demand occurs in the face of lower DIN availability (Brookshire et al., 2005). However, data synthesis has suggested that the catchment N-saturation status is not the only attribute of DON loss, but the variability in catchment characteristics and climate are probably also important factors, and that the evaluation of the changes in the composition and reactivity of DON associated with increased N loading is crucial (Pellerin et al., 2006).

The processing and fate of organic C is fundamentally and synergistically linked to the N cycle (Taylor and Townsend, 2010; Ghosh and Leff, 2013). Lability of DOM influences the tendency of DIN or DON to be the dominant N source for heterotrophic bacterial growth (Manzoni et al., 2010; Ghosh and Leff, 2013). With low N-loading, the ecosystem loss of DOC couples with DON (Aber et al., 1998; Vitousek et al., 2002), as hypothesised by the “*Passive Carbon Vehicle*” theory, and the quality of DOM is static (Brookshire et al., 2007). For chronically N-saturated catchments, however, N-enriched DON loss exceeds and decouples from DOC concentrations (Brookshire et al., 2007; Fang et al., 2009), and has been hypothesised as complying with the “*Stoichiometric Enrichment*” theory (Brookshire et al., 2007).

To our knowledge, there is scarce field evidence resolving the transport mechanisms of DOM and inorganic nutrients simultaneously, especially in montane forest systems subjected to drastic changes in hydrologic phases. In this study, we hypothesised that these kinds of catchments will shift between “*active pipe*” and “*passive pipe*” during low and extreme hydrologic regimes, respectively. Moreover, streams with different discharge per unit area will generate divergent flow paths during extreme events; that the N-saturation state and quality of DOM will be reflected on the respective transport mechanism. That is, DOM quality will not only be a result of the interplay between hydrologic and spatial dimensions but is also an internal force driving the system to export DOM and nutrients passively (coupled) or actively (decoupled).

## 2 Material and methods

### 2.1 Study site

**Fig. 1:**
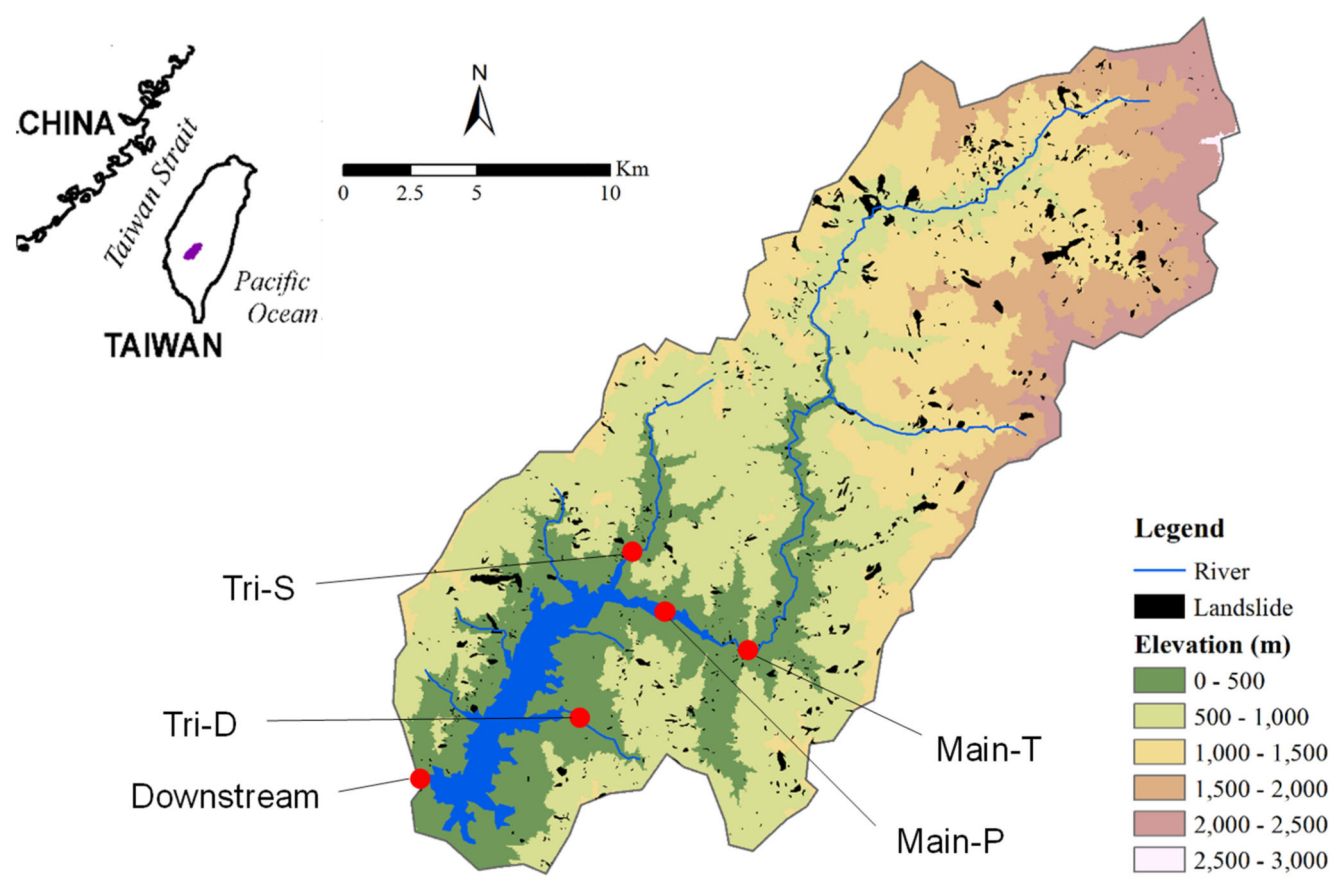
Sampling stations (red circles) within Tsengwen reservoir catchment. Landslides triggered by typhoons from 2004 to 2014 are indicated in black.

Tsengwen Reservoir, (23°14′53″N, 120°32′11″E) with a capacity of 708 million m^3^, is the largest operating reservoir for irrigation, flood regulation, hydropower generation, domestic and recreational use for southern Taiwan (Fig. 1). The mainstream, Tsengwen River, runs 56.2 km from headwater (Wangsuishan Mountain, 2440 m) and has a total catchment area of 481 km^2^ with a mean elevation of 963 m and a mean slope of 54.4%. The whole catchment is characterized by a humid sub-tropical climate with a mean annual temperature of 24.3 °C with 27.7 °C in summer and 17.8 °C in winter (Southern Region Water Resources Office, Taiwan). In southern Taiwan, about 89% of annual rainfall (2525 mm) and 88% of subsequent riverine discharge (17,436 × 10^6^ m^3^) are concentrated between May and October, showing the distinct seasonality to which monsoon frontal rains and typhoons are the main contributors. This distinct seasonality marks the intra-annual dry-wet cycle with drastic differences in hydrologic regimes. For base-flow sampling, two tributary stations, *Shalun* “Tri-S” situated north and *Datung* “Tri-D” situated central-east of the reservoir, one main river station, *Tzjing* “Main-T”, and the reservoir downstream-dam site were sampled (Fig. 1). However, due to limited accessibility during typhoon Matmo, samples from another mainstream station which was about 3 km downstream of Main-T, *Dapu* “Main-P”, was sampled along with Tri-D and Tri-S. During typhoon Soudelor, Tri-D and Main-T were sampled. In general, the two tributary catchments have higher percentages of agricultural lands. Tri-S features steeper slopes and a higher percentage of bare-land area, which is probably due to old landslide scars (Table 1). According to the land-use survey in 2012 (National Land Surveying and Mapping Center, Taiwan), 77% of the total catchment area was made up of forest, while other land-use categories included betel nut plantation (4.3%), bare-land (3.6%), grassland (3%), tea plantation (1.5%), fruit trees (1%), and dry farming (1.1%). In recent years, increased betel nut and tea cultivation has accelerated the clearance of forest cover.

### 2.2 Field sampling and laboratory measurement

Base-flow sampling was conducted biweekly or monthly over the course of 2.5 years, leading to a total N = 108 for low flow and N = 91 for high flow periods. Additionally, typhoon samplings were conducted intensively at 2–3 h intervals during two complete typhoon events, each for 45–50 h (total N = 110). Two typhoons, *Matmo* (moderate scale, 135 km h^−1^, 22–24 Jul 2014) and *Soudelor* (severe scale, 210 km h^−1^, 7–10 Aug 2015) were sampled. Matmo and Soudelor totalled 286.8 mm rainfall in 49 h and 313.4 mm in 51 h, respectively. However, sites were not accessible during the maximum impact period due to a road blockage caused by slump and trees, resulting in a 9-h gap in the Soudelor sampling. The discharge of the mainstream was measured at Main-P Dapu gauging station (Fig. 1). A modified 3-layer TOPMODEL was applied to estimate the discharge of ungauged tributaries following the methods described in Huang et al. (2009). The results were presented as runoff depths (mm d^−1^) by taking the catchment area into account.

At each site, 3 L of free-flowing surface river water were collected from the middle of the bridge using rinsed polyethylene bottles mounted to a set of stainless steel racks. Physical-chemical parameters were measured on site with a multi-parameter water quality meter (Pro Plus, YSI). On the same day, water samples were filtered through muffled and pre-weighed GF/F filters (0.7 μm pore size, Whatman), the filters were oven-dried (60 °C, 72 h) and weighed for total suspended matter (TSM). The filtrate was stored in a freezer (−20 °C) until further water chemistry analyses could be performed. DOC and total dissolved nitrogen (TDN) concentrations were determined via high temperature combustion using an auto TOC/TN analyser with a detection limit of 48 and 50 μg L^−1^ C and N, respectively (Multi N/C ® 3100, Analytik Jena AG). Dissolved inorganic nitrogen $\left(\text{i}.\text{e}.\text{N}{\text{O}}_{3}^{-},\text{N}{\text{O}}_{2}^{-}\;\text{and}\;\text{N}{\text{H}}_{4}^{+}\right)$ and Cl^−^ concentrations were determined by ion chromatography (883 Basic IC plus, Metrohm) with a detection limit of 0.2, 0.2, 0.4, 0.1 μM, respectively. $\text{P}{\text{O}}_{4}^{3-}$ was determined with the standard Molybdenum blue method with a detection limit of 0.01 μM (Parsons et al., 1984). DON was calculated by subtracting DIN from TDN concentrations.

### 2.3 Optical properties of DOM

Extra samplings were conducted for DOM optical properties analysis – two samplings at base-flow (17 April and 15 May) and one after typhoon (15 August 2015) were collected at Tri-D, Tri-S, Main-T; nine samplings during typhoon Matmo (23 and 24 July 2014) were collected at Tri-D and Tri-S; and four samplings during typhoon Soudelor (7–10 August 2015) at Tri-D and Main-T, amounting to a total N = 36. All samples were stored at 4 °C and processed within 48 h. The samples were pre-filtered through GF/A (Whatman, USA) and subsequently through 0.45 μm membrane filters (Pall, USA). Each sample was first scanned for its absorbance at 200–800 nm on a spectrophotometer (Hitachi U-2900); if the absorbance at 254 nm exceeded 0.3 A.U., dilution was conducted for avoidance of inner-filtration (Ohno, 2002). One drop of H_2_SO_4_ (0.3N) was then added to each 15 mL sample to preventing fluorescence quenching by iron (Poulin et al., 2014) before analysing on a fluorospectrometer (Hitachi F-7000). pH is one of the factors influencing the optical properties of DOM. However, the pH range of our typhoon and base-flow DOM samples were alkaline (8.17–9.4), when comparing with studies using pH as a factor to quantify DOM variations (e.g. Yan et al., 2013). The samples are well-buffered due to the abundance of carbonate ions in the river water from Tsengwen catchment (Shih et al. in preparation), thus not expecting a massive change in pH after the acid addition. A 3-d excitation-emission matrix (EEM) was generated via scanning the sample at excitation wavelength between 200 and 450 nm at 5 nm steps and emission wavelengths between 250 and 600 nm at 2 nm steps (Baker, 2001). The value of fresh double-distilled water of the experiment day was subtracted from the sample, and each fluorophore group was identified as each peak appeared on the EEM.

Chromophoric or fluorophoric indices were used as descriptors to characterise DOM; they were derived from various ratios between certain wavelengths or regions of an absorbance spectrum or EEM, respectively, following the calculations and descriptions of Fellman et al., 2010. Absorbance data were first baseline corrected by subtracting the mean absorbance from 680 to 700 nm, and then converted to absorption coefficient (Xu et al., 2016; Helms et al., 2008). Specific UV-absorbance at 254 nm (SUVA_254_, UV absorbance at 254 nm measured in inverse meters divided by the DOC concentration in mg L^−1^) resembles the percent aromaticity (Weishaar et al., 2003); absorption spectral slope ratio (S_R_, the ratio of log transformed absorbance spectra slope at 275–295 nm to that of 350–400 nm) correlates oppositely to the molecular weight (MW) of DOM (Helms et al., 2008). EEM peak A (Ex < 260 nm/Em 440 nm) resembles low MW fulvic acids; peak C (Ex 330–370 nm/Em 430–460 nm) resembles high MW humic acids; peak B (Ex 270–275 nm/Em 304–312 nm) was originated from degraded peptide and is linked to tyrosine-like material; and peak T_280_ (Ex 270–280 nm/Em 330–368 nm) indicates intact proteins or less degraded peptides such as tryptophan (Fellman et al., 2010). Biological index (“BIX”, Em intensity at 380 nm divided by the maximum Em intensity between 420 and 435 nm, at Ex 310 nm, Parlanti et al., 2000), humification index (“HIX”, the sum of the peak area between 435 and 480 nm divided by the sum of the intensities between 300 and 345 nm and 435–480 nm, at Ex 254 nm, Ohno, 2002), fluorescence index (“FI”, the Em intensity at a wavelength of 450 nm to that at 500 nm, measure at Ex 370 nm, McKnight et al., 2001), and the humic- and protein-like DOM pools (sum of peak A and C; peak B and T, divided by the DOC concentration, respectively) were calculated. HIX is positively correlated to the degree of DOM humification; high BIX (>1) indicates autochthonous source and low BIX (<0.6) indicates a dominant allochthonous source; terrestrial or allochthonous DOM has a FI < 1.4 and microbial or autochthonous DOM > 1.9. The regression slopes of DOM descriptors versus discharge (*d*DOM/*d*Q) were calculated from their hysteresis loops, and the positive or negative linear regressions were significant at *P* < 0.05, otherwise were categorized as “chemostatic” (Guarch-Ribot and Butturini, 2016). The hysteresis relationship between discharge and DOM quality was only analysed for the Soudelor samples, as discharge data for the specific subcatchments Tri-D and Tri-S were not available for typhoon Matmo.

### 2.4 Data treatment and statistics

Field data was grouped into three hydrologic phases, namely the low flows, high flows, and typhoon events. The beginning of each low flow period was determined by the weakening of summer monsoon and strengthening of winter monsoon (Central Weather Bureau, Taiwan); and the threshold separating low and high flow period was marked by the simultaneous shift of vertical and horizontal wind shear (Chen et al., 2012). Statistical analysis was performed in SPSS statistical software (IBM, version 21). Each parameter was tested for normal distribution using the Q-Q plot and Shapiro-Wilk test (*P* < 0.05); those which were not normally distributed were log- or log(x + 1)-transformed before applying further parametrical analysis. Data was compared between seasons via one-way analysis of variance (ANOVA) to test the relationship between dependent and independent variables, followed by Tukey's test for post-hoc analysis; *P*-values of ≤0.05 were considered significant. Pearson correlation was conducted on normally distributed variables to test if there was a similar trend between variables.

## 3 Results

### 3.1 Physical-chemical conditions during base-flow and typhoons

**Fig. 2:**
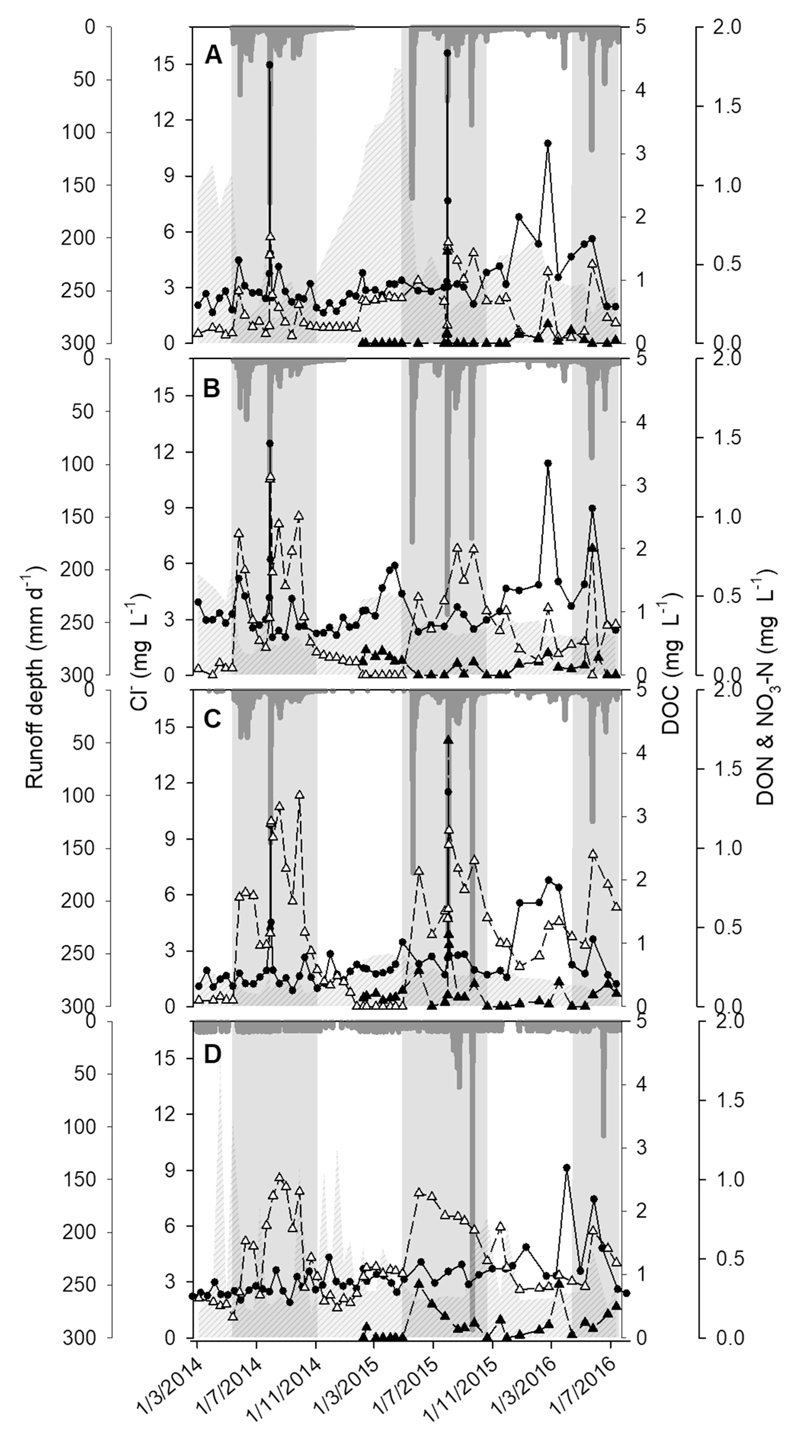
Runoff depth (dark grey bars) and the concentration of Cl^−^ (grey striped area), DOC (●), DON (▴), and NO_3_-N (▵) of (A) Tri-D, (B) Tri-S, (C) Main-T, and (D) downstream during the 2.5-year study period. Grey shaded area = high flow; non-shaded area = low flow.

**Fig. 3:**
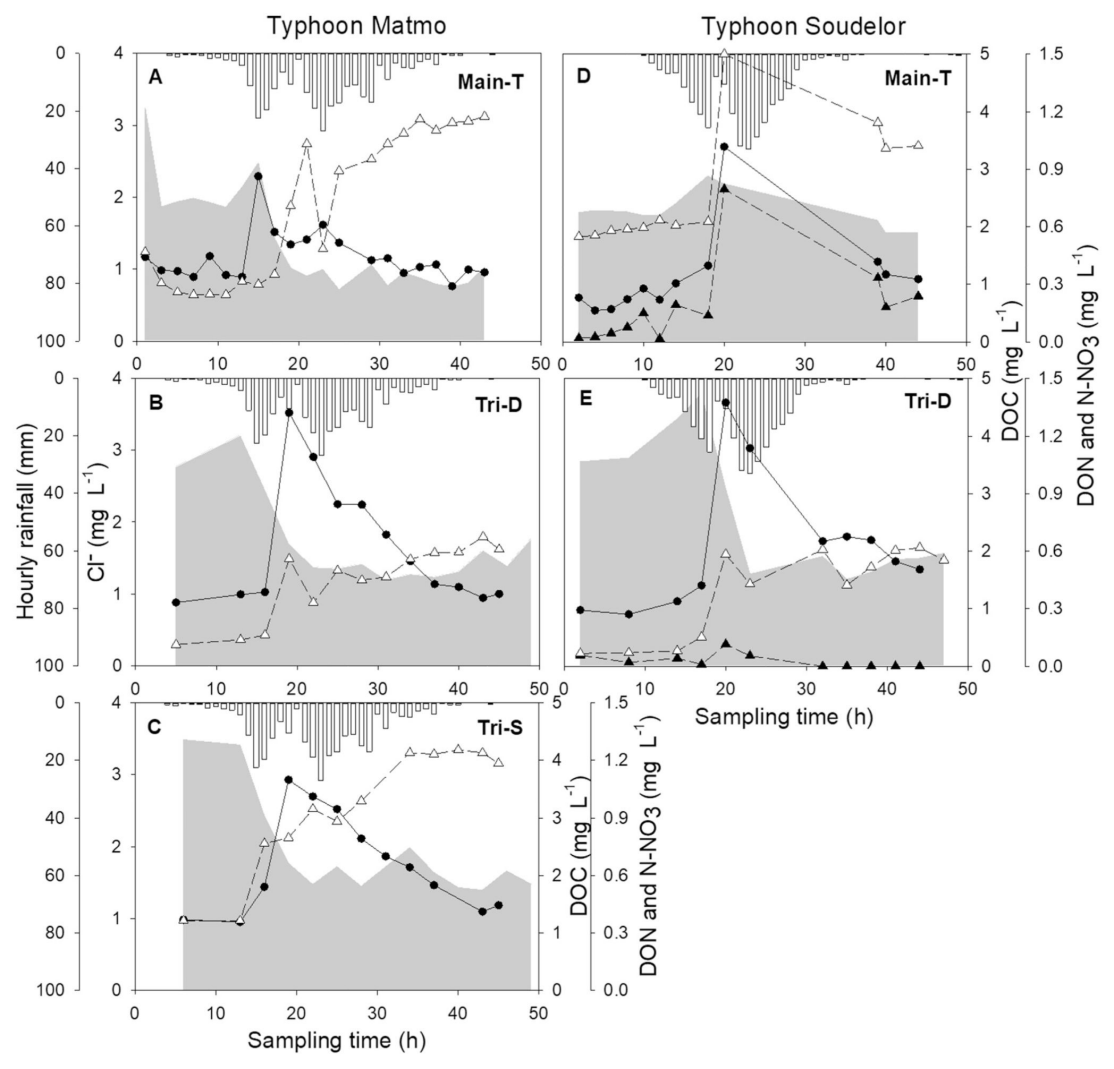
Typhoon sampling during Matmo 2014 (left panels) and Soudelor 2015 (right panels) of hourly rainfall (grey bars), the concentration of Cl^-^ (grey shaded area), DOC (●), DON (▴), and NO_3_-N (▵) at sub-catchment (A)(D) Main-T, (B)(E) Tri-D, and (C) Tri-S during the typhoon period. The sampling from Tri-S was absent during typhoon Soudelor due to logistic difficulties.

The mean runoff depth at each sub-catchment was similar during each hydrologic phase (Fig. 2). During the two typhoons, the mean run-off depth (70.15 ± 76.59 mm d^−1^) of the whole catchment was significantly higher than during the low flow period (0.94 ± 0.98 mm d^−1^) and the high flow period (9.11 ± 11.74 mm d^−1^). The water temperature was higher during high flows (26.7 ± 2.2 °C) than low flows (22.9 ± 3.4 °C) or during typhoon events (25.9 ± 2.7 °C, n = 342, *P* < 0.001), and was negatively correlated with discharge (n = 63, Pearson's *r* = –0.40, *P* = 0.001). The air temperature was 1.1–1.7° lower than the water temperature but maintained the same temporal trend (25.6 ± 1.7 °C, 20.2 ± 3.8 °C, and 24.7 ± 1.8 °C for high, low flows, and typhoon events, respectively). There was no statistical difference in pH between seasons. In this study, the concentration of Cl^−^ was taken as an indication of the relative contribution of groundwater source at each station (Figs. 2 and 3). Both Tri-D and Tri-S showed significantly higher Cl^−^ concentrations during the low flow period than the typhoon event (n = 77 and 64, respectively, *P* < 0.001), however, Cl^−^ concentration was similar between these two hydrologic phases at Main-T.

### 3.2 Temporal variation of organic matter and inorganic nutrient concentrations

Fig. 2 shows the temporal trend of DOC, DON, and nitrate-N for each sampling site during the entire sampling period. The DOC concentrations at tributaries were significantly higher than those at the mainstream throughout our study (n = 80, *P* < 0.001, Table 2). DOC concentrations at each station decreased by about 14–20% from low flows to high flows but increased significantly (about 200%) between high flows and the typhoon event (n = 191, *P* < 0.001, Table 2). However, the concentrations of N-species had different trends at each station. DON at Tri-D decreased by 90% from low to high flows, but Tri-S showed a 21% increase while Main-T increased by 200%; however, none of these increases was significant. During low flows, nitrate-N remained at low concentrations for all three upper streams but was relatively high at the downstream site. Nitrate-N concentrations of Tri-S and Main-T increased by 500% and 450%, respectively, during high flows whereas Tri-D only increased by 43%. In contrast to the response of DON, nitrate-N and phosphate-P values only increased significantly at the tributaries (174%, n = 144, *P* < 0.001; 300%, n = 144, *P* < 0.001) between high flows and typhoon events, but the change was not significant at the Main-T.

During the typhoons, DOC and DON concentrations at each site peaked after the first rainfall vertex (Fig. 3). The maximum recorded DOC concentrations were 4.58, 3.66, and 3.39 mg L^−1^ at Tri-D, Tri-S, and Main-T, respectively; and DON was 0.025 and 0.19 mg L^−1^ at Tri-D and Main-T. Nitrate-N increased rapidly until the rainfall vertex and remained at this level until the end of the event. The concentration of Cl^−^ indicated that there was a stronger dilution process at tributaries due to the sharp decrease of conservative solutes.

Tri-D and Tri-S have 52% more annual DOC yield than Main-T (Table 2); however, Main-T and Tri-S generated similar high magnitude of nitrate-N yield than Tri-D. Moreover, tributaries had about 149% higher phosphate-P yield than the mainstream. This resulted in the higher C:N:P ratio in the Tri-D station than Tri-S or Main-T.

### 3.3 Correlations between multiple organic and inorganic constituents

**Fig. 4:**
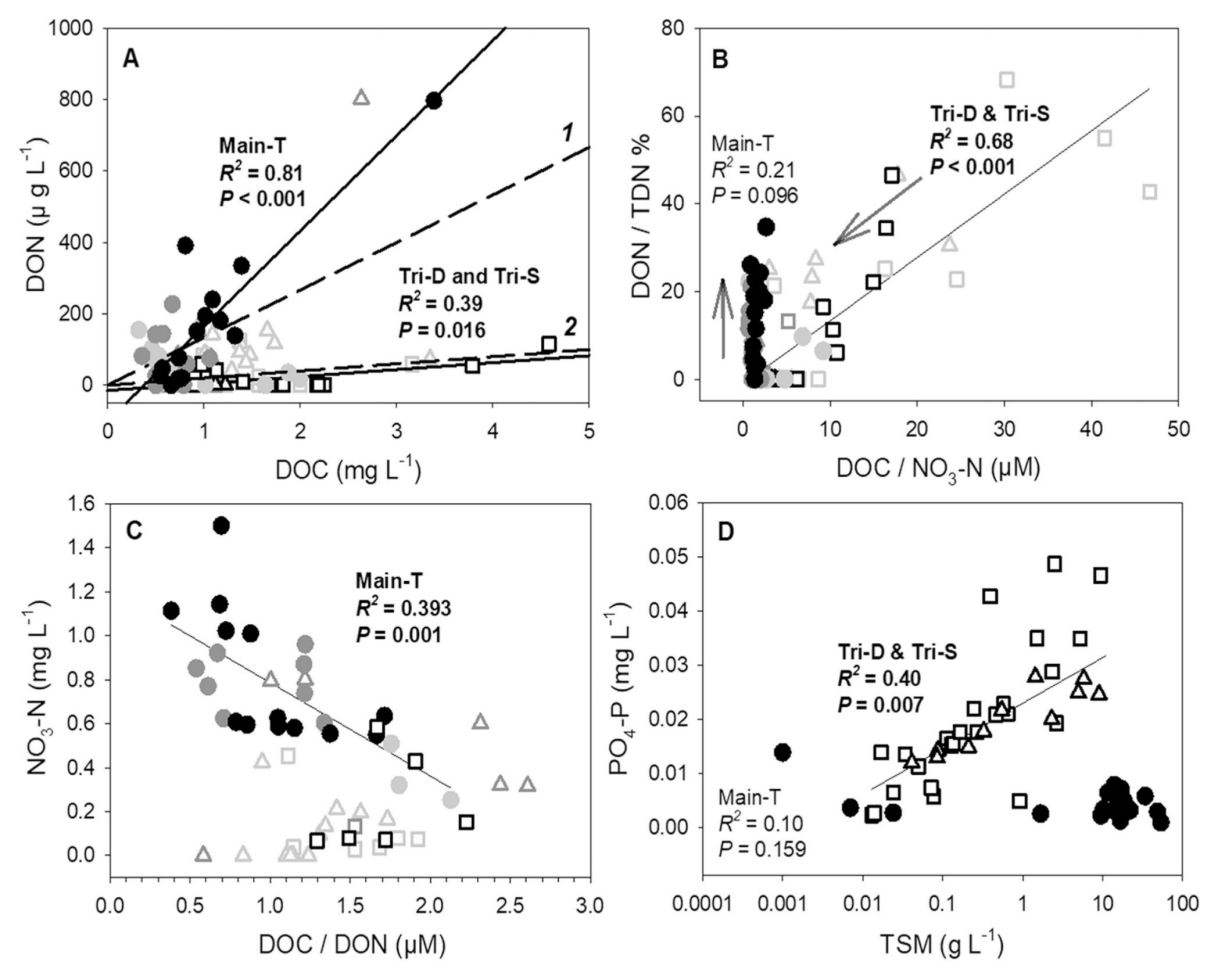
Correlations between variables: (A) Dissolved organic nitrogen (DON) versus carbon (DOC) concentrations. Dash-line 1 = Noland Divide, USA (3220 kg N km^−1^ yr^−1^, H. Van Miegroet, unpublished data); dash-line 2 = low disturbed S. American catchment (<300 kg N km^−1^ yr^−1^, Perakis and Hedin, 2002); (B) DOC:NO_3_-N molar ratio versus DON% in total dissolved organic nitrogen (TDN). The black arrows indicate the time sequence during typhoon sampling; (C) NO_3_-N versus DOC:DON molar ratio (D) concentrations of total suspended matter (TSM) and PO_4_-P. Significant relationships were indicated with bold letters. Filled circles = Main-T; open squares = Tri-D; open triangles = Tri-S; light grey = low flow; dark grey = high flow; and black = typhoon event.

DOC and DON concentrations were significantly correlated at the mainstream during low flows (n = 15, *P* = 0.026, *R*^2^ = 0.33), at the tributaries during high flows (n = 19, *P* < 0.001, *R*^2^ = 0.72), and at both the tributaries and mainstream sites during typhoons (n = 14, *P* = 0.016, *R^2^* = 0.39; n = 14, *P* < 0.001, *R^2^* = 0.80, Fig. 4A). Conversely, nitrate-N and DON concentrations were correlated at the tributaries during low flows (n = 30, *P=* 0.014, *R*^2^ = 0.20), and at the mainstream during high flows (n = 10, *P* = 0.044, *R^2^* = 0.42) and typhoons (n = 14, P < 0.001, *R^2^* = 0.84). The DOC:DON ratio of Main-T showed a chemostatic relationship with discharge during typhoons (*P* = 0.252). However, Main-T had a continuous enrichment of DON% (3.4 to 34.7%) with a static DOC:NO_3_-N ratios during the typhoon; while the DON% at Tri-D decreased (46 to 6%) during low flows and typhoon periods and had a positive correlation with DOC:NO_3_-N (Fig. 4B). The DOC:DON ratio of Main-T correlated well with NO_3_-N (n = 24, *R*^2^ = 0.39, *P* = 0.001) but not for the tributaries; however, Tri-D and Tri-S had their values closer to the regression line of Main-T during the typhoon and the high flows, respectively (Fig. 4C). Phosphate was significantly correlated to DOC (n = 41, *R*^2^ = 0.89, *P* < 0.001) and TSM (*n* = 43, *R*^2^ = 0.57, *P* < 0.001) concentrations in tributaries (Fig. 4D); however, this relationship was not observed in the Main-T samples.

### 3.4 Hydrologic control on DOM optical properties and inorganic solutes during typhoon

**Fig. 5:**
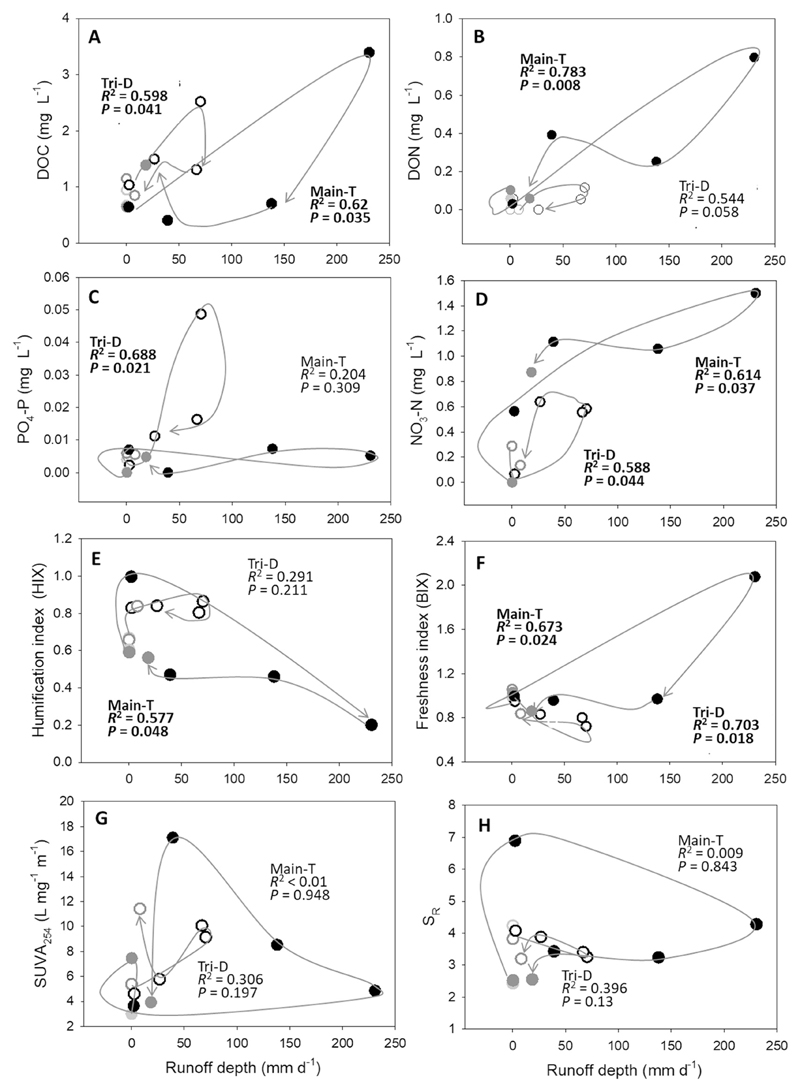
Hysteresis relationships between DOM descriptors and discharge during typhoon Soudelor. (A) DOC, (B) DON, (C) PO_4_-P, and (D) NO_3_-N concentrations; and (E) humification index (HIX), (F) Freshness index (BIX), (G) SUVA_254_, and (H) S_R_. Time sequence is indicated by grey lines and arrows. Linear regression model (*d*DOM/*d*Q) was applied to each sub-catchment; significant relationships were indicated with bold letters. Filled circles = Main-T; open circles = Tri-D; light grey = low flows; dark grey = high flows; and black = typhoon events.

DOC concentrations at Tri-D and Main-T had similar positive (*d*DOC/*d*Q > 0) and clockwise hysteresis loops with discharge, suggesting flushing processes (Fig. 5A). However, a significant C-Q relationship only existed for DON at Main-T station (*d*DON/*d*Q > 0); and for phosphate-P at Tri-D station (*d*PO_4_-P/*d*Q > 0). The C-Q relationships were not significant at Tri-D for DON and at Main-T for phosphate-P concentration (Fig. 5B,C). The positive and clockwise *d*NO_3_-N/*d*Q relationship at Main-T was similar to DON concentrations; however, the positive but counter-clockwise correlation at Tri-D suggested a lagged infiltrating release of nitrate (Fig. 5D).

**Fig. 6:**
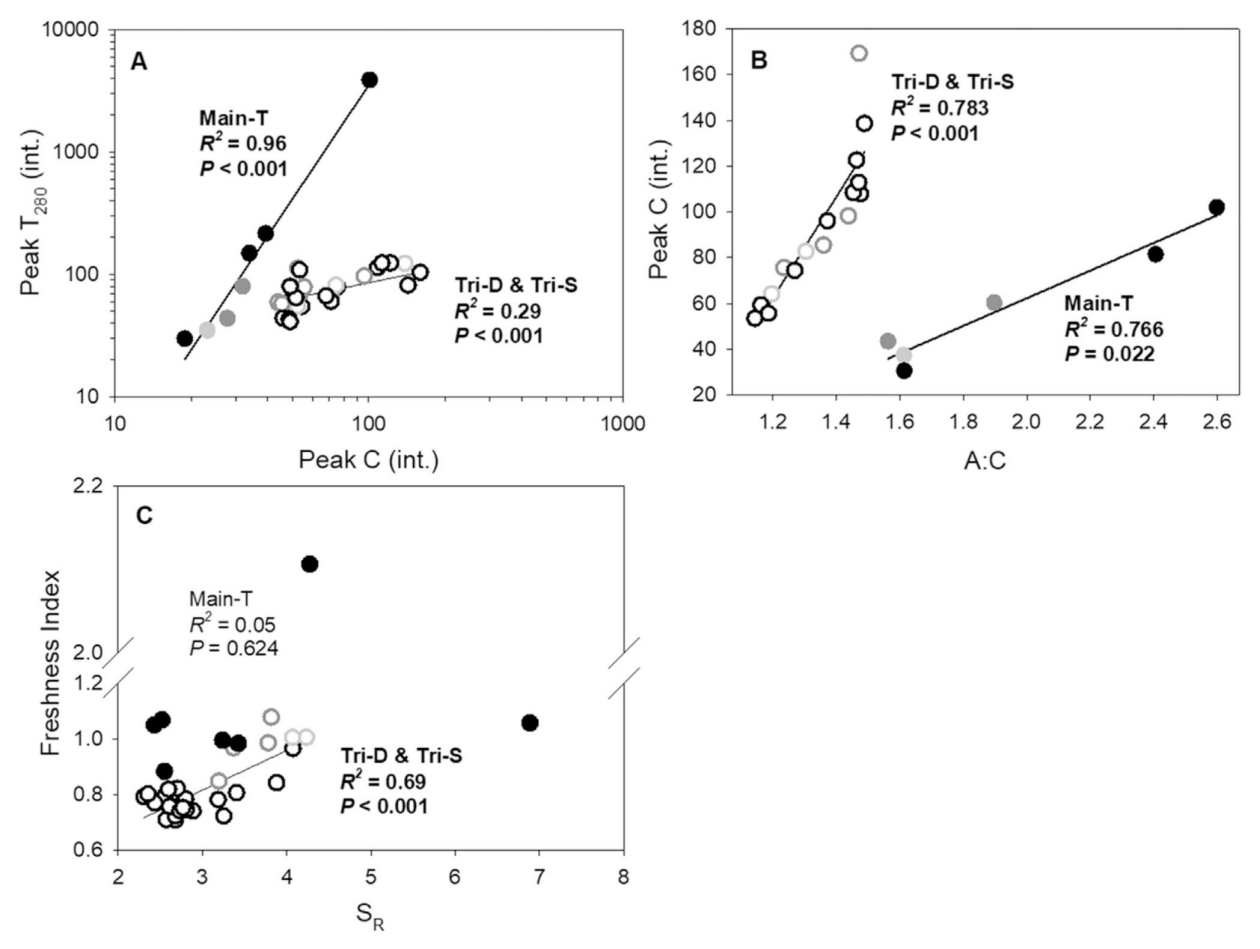
The fluorescence intensity of (A) Peak T_280_ (tryptophan-like) versus Peak C (humic acid-like), (B) Peak C (humic acid-like) to A:C ratio, (C) BIX (freshness index) to S_R_ (molecular size) during each hydrological phase and sampling stations. Significant relationships were indicated with bold letters. Filled circles = Main-T; open circles = Tri-D; light grey = low flow; dark grey = high flow; and black = typhoon event.

Regarding DOM composition, HIX was chemostatic at Tri-D station, while Main-T demonstrated a strong negative correlation (*d*HIX/*d*Q < 0) which suggested a diluting process (Fig. 5E). In contrast, BIX showed a significant enrichment at Main-T, while showing a diluting process at Tri-D (Fig. 5F). The concentration-discharge relationship of SUVA_254_, S_R_, and FI were chemostatic. SUVA_254_ was always high at the end of the events, which could be due to the simultaneous increase of both humic- and protein-like DOM pools (aromatic amino acids, i.e. tryptophan and tyrosine) (Fig. 5G and Table 3). S_R_ increased in the beginning of the typhoon event at Main-T, suggesting a transport of low molecular weight DOM (Fig. 5H). The high value of FI on average, showed the whole Tsengwen catchment was probably dominated by autochthonous DOM, especially during the typhoon periods. The regressions between peak T_280_ and peak C (Fig. 6A) demonstrated Main-T samples were enriched with less degraded aromatic protein (tryptophan-like) and soluble microbial by-products (Ex 250–280 nm/Em < 380 nm) which led to the intensification of peak T_280_ during the proceeding of the typhoon (T_280_:C ratio slope = 3.104). Consequently, the ratio between tyrosine and tryptophan demonstrated an extreme reduction during the typhoon peak discharge (Table 3). On the other hand, Tri-D was enriched with humic-derived substances (Fig. 6B) and tyrosine-like protein material, thus, the T_280_:C ratio had a gentler slope (= 0.525). This resulted in an increase of the average DOM molecular weight and a decrease of DOM freshness for the Tri-D samples (Fig. 6C).

## 4 Discussion

### 4.1 The flow paths of tributaries and mainstream

Tributaries received higher groundwater input than the mainstream regardless of season or discharge as evidenced by the significant fluctuation of Cl^−^ concentration. During the typhoon, the C-Q relationship of DOC, phosphate-P and nitrate-N became positive at the tributaries; however, there was no significant relationship for DON. Moreover, similar to other studies of forested catchments (e.g. Guarch-Ribot and Butturini, 2016), the main source of DOM was derived from the soluble fraction of the surface or subsurface soil organic matter, specifically humic substances and tyrosine. BIX and protein-like DOM pool both showed a dilution response with increasing discharge (Table 3 and Fig. 5F). These results suggested that the Tri-D DOM during the course of typhoons was allochthonous-dominated and highly degraded material. It was probably from the shallow subsurface degraded organic soils; this comparatively deeper and slower flow path resulted in a longer contact time for the storm water to extract OC-rich DOM from the soils than that of the overland flow (Salmon et al., 2001). Furthermore, previous studies showed that the fluctuation of DOC during typhoons was due to the OC-enriched organic topsoils significantly differing from the OC-deficient deep mineral soils in Taiwan (Lee et al., 2013). The flow path of Tri-D switched to deeper soil layers during the descending part of the typhoon hydrograph, which was indicated by other studies where the depleting DOC pool and increasing nitrate-N concentrations were linked to the mineral soil layers (Brady, 1990); as also confirmed by other observations from forest catchments of mountainous island (Zhang et al., 2007).

The mainstream showed a different picture in terms of DOM sources and inorganic nutrients; it was probably preceded by overland runoff owing to the intensive typhoon precipitation. Schmidt et al. (2010) found that in forest soils of a low disturbed mountainous catchment in Taiwan, DON was the dominant N species exporting from the terrestrial system, and that the precipitation intensity plays a crucial role in exporting soil DOC and DON. In our study, this was evidenced from the positive and clockwise C-Q relations for several indicators (i.e. DOC, DON and BIX), which signalled intensive flushing processes (Zhang et al., 2007) at Main-T. Phosphate was the only solute which showed no significant C-Q relationship at the mainstream (Fig. 5C). In the SMRs of Oceania, the main source of phosphate derives from the surface soil as a product of strong erosion processes that are mostly restricted to intensive rainfalls or typhoon events (Lee et al., 2013). The decoupling of phosphate-P and TSM in our typhoon samples suggests that these solids mobilised by erosion process were not the main carriers of phosphate-P at Main-T. Instead, TSM was probably sourced from the re-suspension of riverbed siltation from the massive erosion events of previous typhoon and was therefore P-depleted due to its transporting history. On the other hand, we found contradictory result concerning the low agricultural activity and the high export of nitrate and DON at Main-T catchment. Current studies investigating agricultural impact on either DOC (Wilson and Xenopoulos, 2008; Graeber et al., 2012) or DON export from catchments (Pellerin et al., 2006; Stedmon et al., 2006) show no consistent pattern, which is probably a combined result of the diverse agricultural practices and the subsequent effect on soil processes and the aquatic carbon cycle (Stanley et al., 2011). Graeber et al. (2015) concluded that, DIN correlates well with DON export and the changes of DOM composition in agricultural-impacted streams across climate zones, and the main driver is probably the mechanisms which related to the intensification of agriculture (e.g. soil tillage and fertilization level). Nitrate of the SMRs in Taiwan is presumably derived from the abundant subterranean reservoirs (Lee et al., 2013). However, we found that among sub-catchments in the same drainage system, agricultural percentage is probably not a reliable predictor for the fluvial transport of organic and inorganic matter. The rapid overland flow preceded in Main-T was enriched with freshly-produced and poorly degraded tryptophan-like DOM (Fig. 6A), most likely derived from near-stream sources (Lloyd et al., 2016) such as the riverbed or riparian area (Butturini et al., 2003; Guarch-Ribot and Butturini, 2016). The fact that flow regime changes the DOM composition of Main-T, a supposedly low anthropogenic-impacted catchment, probably explaining the contradictory result between the low percentage of agriculture and the transport of N-enriched DOM in this study. To sum up, the accumulation of the terrestrial DIN pool from agricultural activities (e.g. level of fertilization) or bioactivity on forest floor could provide the potential “source”; while the hydrologic regime largely decides the “accessibility” of this pool entering the aquatic system. However, we also observed divergent relationships between exported inorganic and organic constituents in Tri-D and Main-T catchments, and we hypothesised it was probably initiated by their differences in N-saturation status as a result of the dominant flow path.

### 4.2 Transport mechanisms of DOM

The regression slope between DON versus DOC indicates the N-saturation status of a catchment (Brookshire et al., 2007). In this study, N-saturation status of both Tri-D and Main-T catchment was strongly controlled by the hydrologic regime, shifting both catchments between unsaturated and N-saturated conditions during low and high flows, respectively (Fig. 4A). Estimated by the annual nitrate-N yield, Tri-D catchment has a lower degree of N-saturation, which was similar to a relative pristine S. America catchment (<300 kg N km^−2^ yr^−1^, Perakis and Hedin, 2002). During low flows and typhoons at the two tributaries, decreasing DOC:NO_3_-N ratio is coupled with DON% as the discharge increased (Fig. 4B), demonstrating the constant TDN loss rate but shifted in the N forms which agrees well with the *Passive Carbon Vehicle* hypothesis (Brookshire et al., 2007). This change can be attributed to the shift of dominance between humic- and protein-like DOM (e.g. Datung during typhoon Matmo, Table 3); however, considering the size of humic- and protein-like DOM pool were similar throughout typhoon Soudelor, the fluctuation of DON was probably a result of the source alternating between fulvic- (lower DOC:DON ratio, lower MW, more soluble) and humic acid-like DOM (higher DOC:DON ratio, higher MW, less soluble) (Stevenson, 1994). The fast increase DOC relative to DON (Fig. 4A), and the negative relationship between molecular weight and DOM freshness (Fig. 6C) supported this point; these trends were similar to other humic-dominant temperate catchments (e.g. Guarch-Ribot and Butturini, 2016).

Previous plot-scale fertilization studies and synthesis showed a strong negative correlation between NO_3_-N and DOC:DON in high N-loaded catchments (McDowell et al., 2004; Pregitzer et al., 2004; Brookshire et al., 2007), and it has been regarded as an integrator of soil C:N and N status of the catchment (e.g. Goodale et al., 2000; Campbell et al., 2000; Brookshire et al., 2007). Indeed, Main-T had a high annual N yield and the lowest C:N yield ratio, showed a constant negative relationship between NO_3_-N and DOC:DON across hydrologic phases (Fig. 4C). Interestingly, while Tri-S had a typical DOC-rich tributary characteristic during low flows, however, as its DOC:NO_3_-N ratio decreased to <10 during high flows (Fig. 4B), the N status became closer to that of the Main-T which is probably saturated (Fig. 4C). This behaviour indicated the ecosystem stoichiometry switched to a C-limited scenario relative to N (Taylor and Townsend, 2010) as discharge increased and more nitrate-N infiltrates from the riparian soil into the stream water (Lee et al., 2013).

The regression slope of DON versus DOC at Main-T during typhoon was closer to that of Noland Divide, USA (3220 kg N km^−2^ yr^−1^, H. Van Miegroet, unpublished data), nevertheless, the yield of Main-T was about half that of the Noland Divide. The overland flow during typhoon supplied Main-T with highly N-enriched DOM (Fig. 6A). Main-T showed a continuous enrichment of DON within the TDN pool during typhoon, while its DOC:NO_3_-N ratio remained chemostatic with increasing discharge (Fig. 4B). It is crucial to point out, this increase of DON loss was additive (i.e. relatively constant nitrate-N loss rate with increasing TDN losses) which agrees well with the *Stoichiometric Enrichment* theory (Brookshire et al., 2007). Low DOC:NO_3_-N ratio can be interpreted as the increasing C limitation of microbial anabolism, and that the stoichiometric controls over heterotrophic activity regulate nitrate-N build-up (Taylor and Townsend, 2010). However, laboratory experiments showed that the microbial immobilization of N into DOM is probably bypassed upon receiving increased amount of nitrate (Dail et al., 2001). Studies conducted in (sub-)tropical forest have reported similar high DON concentration in spite of a low agricultural/settlement percentage (e.g. Schmidt et al., 2010; Jacobs et al., 2017). In fact, hydrophilic amine-rich compounds exuded by mycorrhizae (Aitkenhead-Peterson et al., 2003) may be directly introduced to the DON flux and lower the DOC:DON ratio. This is a probable scenario, considering the overland flow through the riparian forest floor at the Main-T catchment. This was evidenced from the low molecular weight compounds on the rising limb of the hydrograph (Fig. 5H), which had a low SUVA_254_ value (Fig. 5G). Other abiotic pathways are also possible mechanisms, including DIN reacting with DOC to re-synthesize N-enriched DOM (Fang et al., 2009), saturation of sorption sites (Brookshire et al., 2007), and the shift of DOM composition to neutral molecules (Kaiser and Zech, 2000). The concentrated nitrate in an N-saturated system can generate a flux of highly reactive nitrite, which reacts quickly (within <15 min, Dail et al., 2001) with soil-derived refractory DOM and generate DON-rich compounds (Davidson et al., 2003; Fang et al., 2009). According to data collected along Main-T, the top soil of Tsengwen catchment is organic-rich (O-horizon C_org_ = 40.2–42.75%; A-horizon = 5–9.41%) and of acidic character (pH = 5.2–5.7); which is in line with the conditions required for the abiotic reaction (Dail et al., 2001). We only have limited data on the soil properties in this region, however, this hypothesis merits further investigation. On the other hand, a shift of DOM composition was reflected in the tremendous increase in protein-like DOM pool at peak discharge (Table 3). These small and neutral aromatic amino acids molecules probably have limited sorption ability to mineral soil (Kaiser and Zech, 2000; Brookshire et al., 2007) and thus are more likely to be exported to the stream rather than retaining in the terrestrial system.

## 5 Conclusion

This study suggests that the “*pulse-shunt*” effect in terms of DOM transport is prominent in the mountainous streams in Taiwan due to the drastic hydrologic regimes. However, during extreme hydrologic events, passive and N-enriched transport of DOM occurred even for adjacent sub-catchments inside the same drainage system. We point out the key factor might be the N-saturation status caused by the specific flow paths triggered, which could even obscure the effect of land-use. Moreover, dominated by humic- and protein-associated DOC pool, Tri-D and Main-T of this study showed low and high degree of N-affinity, respectively. We further hypothesise that, not only does the saturation status of N affect the DOM enrichment reaction, but the quality of the DOC also plays a decisive role on the affinity between DIN and DON. The DOM transport mechanism during extreme hydrologic events thus probably could not be attributed to a simple conservative process. Instead, the exported DOM might have resulted in divergent fates within the aquatic system depending on the mechanisms being triggered.

Our results provide key insights for riverine management strategies. The most important message was the enhancement of spatial heterogeneity (e.g. DOM quality, N-saturation status) during extreme hydrologic periods. Although the N-saturation status of a catchment can alternate between hydrologic phases, we also observed that the DON loss pattern in the chronical N-saturated system remained constant throughout different phases. This probably implies that the catchments have limited plasticity in terms of N-loading relative to DOC concentration. We highlighted the importance of taking the characteristics of individual catchment into account when developing a management strategy, and the need for integrating the hydrologic cycles into the scheme.

Future studies should focus on investigating abiotic pathways transporting fluxes of organic and inorganic C and N in (sub-)tropical small mountainous catchments, since our study suggested the N-saturation state in this region might play a decisive role. For catchments dominated by different flow paths during extreme hydrologic events, clarifying the soil C and N capital in each sub-catchment will be important for estimating future organic matter and nutrient output. Lastly, considering climate change, it is also crucial to continue studying the combined effects of environmental conditions (e.g., discharge, temperature and irradiation, etc.) on processing in-stream DOM, the effects of N-loading, and the quality of DOM during different hydrologic conditions. To understand the mechanisms behind nutrient and organic matter dynamics under an enhanced hydrologic cycle, will be an important task for reservoir catchment management in light of a changing climate.

## Figures and Tables

**Table 1:** Characteristics of each studied sub-catchment, and the percentage of different land-use categories estimated from land-use maps. The remaining land use types were categorized as *other*.

Station	Elevation range (m)	Mean elevation (m)	Area (km^2^)	River length (km)	Mean slope (%)	Agri-culture (%)	Forest (%)	Bare-land (%)	Built-up (%)	Other (%)
Tri-D	249–1120	625.7	11.1	6.65	52.2	13.9	83.3	1.6	0.6	0.6
Tri-S	236–1282	737.2	40.1	11.59	50.5	22.9	70.3	5.3	1.3	0.2
Main-T	242–2610	1260.7	274.1	43.07	56.5	6.9	87.7	3.0	1.0	1.4
Downstream	–	–	497.9	–	–	10.8	80.3	3.5	1.2	4.2

**Table 2:** Mean, standard deviation (Stdev), maximum, and minimum of DOC, DON, nitrate-N, and phosphate-P concentrations (mg L^−1^) during low/high flows, and typhoon events observed at three upper-stream stations Tri-D, Tri-S, Main-T and a downstream station of the dam; their mean annual yield (kg km^−2^ yr^−1^) and C:N:P ratio of the annual yield. Letters indicate significant differences between hydrological phases for each sub-catchment (*P* < 0.05).

		Tri-D	Tri-S	Main-T	Downstream
		Mean ± Stdev	Mean ± Stdev	Mean ± Stdev	Mean ± Stdev
		Max/min	Max/min	Max/min	Max/min
Low flow	DOC	1.00 ± 0.56a	1.18 ± 0.53a	0.73 ± 0.48ab	1.03 ± 0.45
		3.16/0.48	3.35/0.63	1.99/0.31	2.69/0.65
	DON	0.02 ± 0.04a	0.08 ± 0.05a	0.04 ± 0.04a	0.05 ± 0.09
		0.12/0.00	0.16/0.00	0.15/0.00	0.34/0.00
	NO_3_-N	0.16 ± 0.12a	0.10 ± 0.12a	0.16 ± 0.18a	0.32 ± 0.13
		0.45/0.00	0.42/0.00	0.54/0.00	0.7/0.00
	PO_4_-P	0.0047 ± 0.0026a	0.0022 ± 0.0015a	0.0019 ± 0.0016a	0.0033 ± 0.0036
		0.0115/0.0012	0.005/NA	0.0056/NA	0.018/0.0002
High flow	DOC	0.86 ± 0.26a	0.94 ± 0.44a	0.54 ± 0.20a	0.89 ± 0.20
		1.65/0.56	2.64/0.60	1.06/0.25	1.42/0.56
	DON	0.002 ± 0.007a	0.097 ± 0.25a	0.08 ± 0.07ab	0.13 ± 0.1
		0.02/0.00	0.80/0.00	0.23/0.00	0.34/0.00
	NO_3_-N	0.23 ± 0.16a	0.50 ± 0.28b	0.72 ± 0.28b	0.66 ± 0.22
		0.57/0.00	1.00/0.00	1.33/0.23	1.01/0.27
	PO_4_-P	0.0061 ± 0.0038a	0.005 ± 0.004a	0.004 ± 0.003ab	0.0068 ± 0.012
		0.015/0.0015	0.015/0.0005	0.014/0.0005	0.057/0.001
Typhoons	DOC	1.99 ± 1.07b	1.98 ± 0.87b	1.05 ± 0.7b	–
		4.58/0.9	3.66/1.12	3.39/0.55	
	DON	0.025 ± 0.036a	–	0.19 ± 0.21b	–
		0.12/0.00		0.8/0.00	
	NO_3_-N	0.42 ± 0.22b	0.82 ± 0.40c	0.76 ± 0.32b	–
		0.67/0.07	1.25/0.00	1.5/0.26	
	PO_4_-P	0.019 ± 0.013b	0.015 ± 0.009b	0.006 ± 0.003b	–
		0.049/0.002	0.0278/0.0015	0.014/0.0026	
Annual yield	DOC	3089.0 ± 985.8	3148.6 ± 433.0	2047.5 ± 522.4	2146.3 ± 715.7
	NO_3_-N	765.8 ± 177.9	1686.9 ± 523.8	1624.9 ± 294.2	1843.3 ± 1432.0
	PO_4_-P	28.1 ± 12.2	20.2 ± 7.8	9.7 ± 1.5	7.1 ± 2.2
C:N:P ratio		283.3:70.2:1	401.3:215:1	542.6:430.6:1	773.1:664:1

**Table 3:** Chromophoric and fluorophoric property descriptors of dissolved organic matter (DOM) for samples collected during base-flow (17 April, 15 May, and 15 August 2015), typhoon Matmo (23–24 July 2014), and Soudelor (7–10 August 2015) in Tsengwen catchment.

Station	Date (ddmmyyyy)	Time (h)	DOC (mg L^−1^)	SUVA^a^	S_R_^b^	A:C^c^	B:T^d^	FI^e^	BIX^f^	HIX^g^	Humic-like^h^	Protein-like^i^
Datung	23042014	25	2.81	7.09	2.69	1.46	1.27	1.77	0.73	0.82	107.54	89.96
Datung	23042014	28	2.80	6.50	2.57	1.48	1.36	1.79	0.71	1.12	95.36	65.62
Datung	23042014	31	2.28	7.70	2.68	1.44	1.62	1.85	0.72	0.87	105.04	75.59
Datung	23042014	34	1.82	6.74	2.61	1.30	1.63	2.00	0.76	1.32	104.63	89.46
Datung	23042014	37	1.42	8.17	2.81	1.24	1.72	2.06	0.79	0.72	119.04	140.62
Datung	24042014	40	1.37	7.16	2.65	1.20	1.75	2.15	0.81	1.61	102.99	124.74
Datung	24042014	43	1.18	26.70	2.68	1.47	1.28	1.65	0.71	0.98	354.49	203.36
Datung	24042014	46	1.25	6.96	2.31	1.16	1.75	2.21	0.79	2.32	102.68	94.28
Datung	24042014	49	1.42	5.68	2.56	1.09	1.83	2.33	0.80	2.16	80.30	115.03
Shalun	23042014	25	3.15	5.95	2.81	1.51	1.33	1.76	0.75	0.87	96.89	91.25
Shalun	23042014	28	2.64	6.32	2.74	1.45	1.55	1.84	0.74	0.76	100.76	109.41
Shalun	23042014	31	2.33	7.61	3.19	1.47	1.34	1.84	0.78	0.72	119.66	123.90
Shalun	23042014	34	2.14	6.94	2.77	1.37	1.63	1.84	0.75	0.75	106.46	118.98
Shalun	23042014	37	1.83	6.86	2.60	1.27	1.77	2.05	0.82	0.81	92.30	123.30
Shalun	24042014	40	*NA*	*NA*	2.44	1.36	1.72	1.86	0.77	0.80	*NA*	*NA*
Shalun	24042014	43	1.37	16.54	2.89	1.49	1.39	1.75	0.74	0.84	251.82	213.84
Shalun	24042014	46	1.48	7.42	2.35	1.19	1.75	2.48	0.80	0.69	82.26	145.93
Shalun	24042014	49	1.19	8.48	2.70	1.14	1.53	2.32	0.82	0.51	96.32	230.76
Datung	17042015	*NA*	0.94	5.32	4.23	1.51	1.11	1.82	1.01	0.67	121.94	120.01
Datung	15052015	*NA*	1.14	5.37	3.82	1.43	1.15	1.81	1.08	0.66	107.26	120.44
Datung	07082015	1	1.03	4.61	4.08	1.97	1.10	2.10	0.97	0.83	138.08	118.76
Datung	08082015	22	2.52	9.13	3.25	1.88	0.94	1.69	0.72	0.86	182.42	80.02
Datung	09082015	46	1.30	10.06	3.41	1.81	1.12	1.80	0.81	0.80	163.05	128.63
Datung	10082015	70	1.49	5.77	3.88	1.74	1.30	1.90	0.84	0.84	100.15	84.25
Datung	15082015	*NA*	0.85	11.41	3.20	1.93	1.01	1.85	0.85	0.84	184.69	162.18
Shalun	17042015	*NA*	0.98	9.26	4.07	1.49	1.01	1.85	1.01	0.69	132.99	112.88
Shalun	15052015	*NA*	0.95	8.29	3.37	1.55	0.95	1.73	0.97	0.71	191.08	138.79
Shalun	15082015	*NA*	0.97	10.40	3.78	2.22	1.00	1.89	0.99	0.67	174.61	231.07
Tzjing	17042015	*NA*	0.67	2.99	2.43	1.61	1.26	1.90	1.05	0.60	90.18	117.72
Tzjing	15052015	*NA*	0.64	7.45	2.52	1.56	1.24	1.85	1.07	0.59	111.14	153.36
Tzjing	07082015	1	0.64	3.63	6.89	1.61	1.29	3.62	1.06	1.00	77.00	106.10
Tzjing	08082015	22	3.39	4.86	4.28	8.14	0.27	1.75	2.11	0.20	271.47	1456.34
Tzjing	09082015	46	0.70	8.52	3.24	2.60	0.67	2.23	1.00	0.46	201.80	513.09
Tzjing	10082015	70	0.40	17.10	3.43	2.41	1.20	2.23	0.98	0.47	288.13	817.70
Tzjing	15082015	*NA*	1.39	3.93	2.55	1.90	1.48	2.18	0.88	0.56	66.29	142.32

Time is the sampling time series of a typhoon sampling campaign.

a SUVA = specific ultraviolet absorbance.

b S_R_ = absorption spectral slope ratio.

c A:C = fulvic acid to humic acid fluorescence intensity ratio.

d B:T = tyrosine to tryptophan fluorescence intensity ratio.

e FI = DOM fluorescence index.

f BIX = DOM freshness index.

g HIX = DOM humification index.

h Humic-like = sum of peak A and C divided by DOC.

i Protein-like = sum of peak B and T divided by DOC.

## Highlights

- We investigated the interaction between organic and inorganic solutes during typhoons.
- Lateral transport during typhoons enhances local catchment feature.
- Flow paths determined the transport mechanisms between DOM and inorganic nutrients.
- DOM quality and N loading are internal forces driving riverine system to be *active* or *passive pipe*.
